# Circulating Tumor Cell Detection during Neoadjuvant Chemotherapy to Predict Early Response in Locally Advanced Oropharyngeal Cancers: A Prospective Pilot Study

**DOI:** 10.3390/jpm12030445

**Published:** 2022-03-11

**Authors:** Arnaud Gauthier, Pierre Philouze, Alexandra Lauret, Gersende Alphonse, Céline Malesys, Dominique Ardail, Léa Payen, Philippe Céruse, Anne-Sophie Wozny, Claire Rodriguez-Lafrasse

**Affiliations:** 1 Laboratory of Cellular and Molecular Radiobiology, UMR CNRS5822/IP2I, Lyon-Sud Medical School, Univ Lyon 1, Lyon University, 69921 Oullins, France; arnaud.gauthier@univ-lyon1.fr (A.G.); pierre.philouze@chu-lyon.fr (P.P.); alexandra.lauret@univ-lyon1.fr (A.L.); gersande.alphonse@univ-lyon1.fr (G.A.); celine.malesys@univ-lyon1.fr (C.M.); dominique.ardail@univ-lyon1.fr (D.A.); philippe.ceruse@chu-lyon.fr (P.C.); anne-sophie.wozny@univ-lyon1.fr (A.-S.W.); 2 Department of Biochemistry and Molecular Biology, Lyon-Sud Hospital, Hospices Civils de Lyon, 69310 Pierre-Bénite, France; lea.payen-gay@chu-lyon.fr; 3 Department of OtoRhinoLaryngology Head and Neck Surgery, Croix-Rousse Hospital, Hospices Civils de Lyon, 69004 Lyon, France

**Keywords:** circulating tumor cells, predictive biomarker, HNSCC

## Abstract

Patients with locally advanced oropharyngeal carcinoma treated with neoadjuvant chemotherapy are reassessed both radiologically and clinically to adapt their treatment after the first cycle. However, some responders show early tumor progression after adjuvant radiotherapy. This cohort study evaluated circulating tumor cells (CTCs) from a population of locally advanced oropharyngeal carcinoma patients treated with docetaxel, cisplatin, and 5-fluorouracil (DCF) induction chemotherapy or DCF with a modified dose and fractioned administration. The counts and phenotypes of CTCs were assessed at baseline and at day 21 of treatment, after isolation using the RosetteSep^TM^ technique based on negative enrichment. At baseline, 6 out of 21 patients had CTCs (28.6%). On day 21, 5 out of 11 patients had CTCs (41.6%). There was no significant difference in the overall and progression-free survival between patients with or without CTCs at baseline (*p* = 0.44 and 0.78) or day 21 (*p* = 0.88 and 0.5). Out of the 11 patients tested at day 21, 4 had a positive variation of CTCs (33%). Patients with a positive variation of CTCs display a lower overall survival. Our findings suggest that the variation in the number of CTCs would be a better guide to the management of treatment, with possible early changes in treatment strategy.

## 1. Introduction

Head and neck squamous cell cancer (HNSCC) is the sixth most common cancer worldwide [1]. Current therapeutic strategies are multimodal and use either a combination of surgery followed by radiochemotherapy, neoadjuvant chemotherapy followed by radiotherapy, or radiochemotherapy, depending on the tumor location and stage. These strategies have not demonstrated any superiority to date, and locoregional recurrences and/or metastases lead to therapeutic failure with a less than 50% overall survival (OS) at 5 years. Moreover, the onset of metastasis within 12 months following diagnosis is responsible for nearly 88% of deaths [2]. 

Circulating tumor cells (CTCs) represent a heterogeneous population with wide plasticity and include epithelial cancer cells, cells in the process of epithelial-to-mesenchymal transition, mesenchymal cells, and cancer stem cells (CSCs). CSCs are demonstrated to be responsible for self-renewal and tumor growth in HNSCC [3,4]. In addition, the number of circulating CTCs is correlated with poor prognoses in lung, colorectal, prostate, and breast cancers [5,6,7,8,9].

In HNSCC, and in oropharyngeal carcinoma, few studies have explored the role of CTCs before, during, or after treatments. Despite CTCs having been found in 18% to 33% of HNSCC patients [10,11], their impact on progression-free survival (PFS) and OS remains to be established. Some studies have reported that the presence of CTCs correlates with a poor prognosis [11,12,13], i.e., lower PFS and OS; however, other studies did not find any correlation [10,14]. Therefore, further investigation is needed to determine if the identification and numeration of CTCs could help with the management of patients with oropharyngeal carcinoma. 

Patients with locally advanced oropharyngeal carcinoma who are treated with neoadjuvant chemotherapy are currently reassessed both radiologically and clinically after the first cycle of chemotherapy. Patients with a response of more than a 50% response are referred for adjuvant radiotherapy with or without surgery, whereas patients with less than a 50% response or with tumor progression are directed to a palliative chemotherapeutic strategy.

Currently, except for HPV-driven oropharyngeal carcinoma [15,16], there are no biological markers to identify the response to neoadjuvant chemotherapy upon reassessment. Furthermore, some responders at clinical and radiological re-evaluation show early tumor progression after the end of adjuvant radiotherapy.

In this prospective pilot study, 21 patients with locally advanced oropharyngeal carcinoma treated with neoadjuvant chemotherapy were enrolled, and the evolution of CTCs during treatment was explored, both in terms of their cell number and morphological characteristics. Our primary objective was to define whether the number of CTCs before and after the first cycle of neoadjuvant chemotherapy could be a predictive biomarker of therapeutic response. The secondary objective was to determine whether a variation in the number of CTCs between the beginning and the end of the first cycle of neoadjuvant chemotherapy could be predictive of survival.

## 2. Materials and Methods

Study population and sample collection. Twenty-one patients displaying a histologically proven squamous cell carcinoma from the oropharynx were recruited between May 2016 and November 2018 from the Head and Neck Department at Croix-Rousse Hospital (Lyon, France). Tumors were not resectable. The HPV status was obtained by PCR on a tissue biopsy analyzed by the HPV DNA test Clinical^®^ Array Human Papillomavirus Genomica (R-Biopharm, Lyon, France). All HPV-positive patients were p16 positive, except one who was p26 positive. According to our therapeutic protocol, the patients were treated with either neoadjuvant docetaxel, cisplatin, and 5-fluorouracil (DCF) or with docetaxel, cisplatin, 5-fluorouracil, with modified dose and fractioned administration (mDCF) chemotherapy. Some patients later received adjuvant radiotherapy. This study (NCT02714920) was conducted in compliance with French legislation and was approved by the local independent ethics committee in November 2015. Written consent was obtained from each patient. The patients were followed up for 24 months, and the last follow-up was conducted in November 2020. Blood samples were collected from every patient at baseline, i.e., before treatment. On day 21, blood samples were collected only from patients who received DCF chemotherapy due to the schedule of the chemotherapy administration. Blood from one patient who received mDCF chemotherapy was also collected on day 21. The patients’ characteristics are summarized in Table 1.

Classification of patients and change in CTCs. Patients were clinically stratified into early responders or early nonresponders according to their clinical response at 4 months follow-up. The group of responders corresponded to clinical and radiological RECIST remission, while the nonresponders corresponded to disease progression. Changes in CTC number were classified into two categories, positive variation and no positive variation (i.e., stable and negative variation). An absence of CTCs at baseline compared with a presence of CTCs at day 21 was considered as a positive variation. The presence of CTCs at baseline compared with an absence of CTC at day 21 was considered as a negative variation. An absence of CTC at baseline and day 21 was considered as stable. A positive CTC count at baseline and day 21 with an increase in CTC was considered as a positive variation, whereas a decrease was considered as a negative variation.

Isolation of CTCs by RosetteSep^TM^. Blood samples were collected in two EDTA tubes of 10 mL and centrifuged in a 50 mL tube at 1200× *g* for 10 min at room temperature. Plasma was then replaced by phosphate-buffered saline (PBS) at an equivalent volume without mixing. A small volume of residual plasma was left on the surface of the red blood cells to avoid collecting CTCs at the interface. The sample was then incubated with the RosetteSep reagent (Stemcell Technologies, Vancouver, Canada) [17] at 50 µL/mL for 30 min at room temperature under slight agitation. Thereafter, the cellular separation was achieved in SepMate 50 mL tubes containing 17 mL of Lymphoprep density gradient medium (Stemcell Technologies). Samples were centrifuged at 1200× *g* for 20 min at room temperature. The upper phase was transferred to a 50 mL tube and reconstituted to 50 mL with PBS with 2% fetal bovine serum (FBS). After centrifugation at 1200× *g* for 10 min at room temperature, the cell pellet was rinsed twice with 50 mL of PBS and 2% FBS. Finally, the enriched cellular suspension was resuspended in 2 mL of PreservCyt (Hologic, Marlborough, MA, USA) and transferred to a cryotube for storage at 4 °C until analysis.

**Figure 1:**
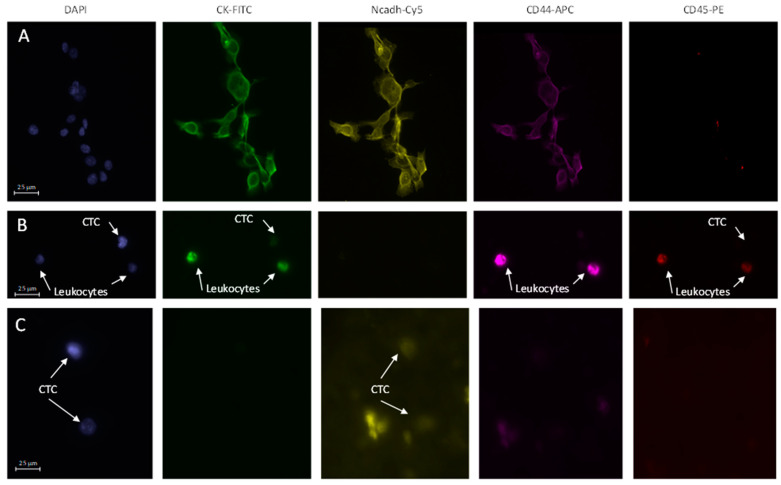
Immunofluorescence stainings (X20): (**A**) SQ20B-CD44+ cancer stem cells expressing cytokeratin, N-cadherin and CD44; (**B**) Representative CTC observed in patient #12, expressing cytokeratin at baseline; (**C**) Representative CTCs observed in patient #1, expressing N-Cadherin at baseline.

Detection of CTCs. The cells stored in the PreservCyt were cytospined on a slide at 18 g for 4 min at room temperature. A droplet of blocking solution (100 µL; PBS with 0.1% bovine serum albumin (BSA), 1% FBS) was dropped onto the slide. After 30 min incubation at room temperature, blocking solution was replaced by 100 µL antibody solution (PBS, 0.1% BSA, 1% FBS), 1:100 anti-cytokeratin-FITC (Miltenyi Biotec, 130-080-101), 1:100 anti-CD44-APC (Miltenyi Biotec, 130-113-338), 1:100 anti-CD45-PE (Miltenyi Biotec, 130-110-632), 1:100 anti-N-Cadherin-Cy5 (Abcam, Cambridge, UK) and 0.1 mg/mL of 4′,6-diamidino-2-phenylindole (DAPI) (Sigma-Aldrich, St Louis, MO, USA). The slide was then maintained at 4 °C overnight. The next day, the solution containing antibodies was removed and 100 µL of 1:1000 AlexaFluor 594 anti-mouse antibody was added and incubated for one hour in the dark. Then, after four washes with 200 µL PBS followed by 5 min of drying at room temperature, the slide was mounted under a coverslip with Fluoromount (Sigma-Aldrich) and polymerized overnight at room temperature before analysis by fluorescence microscopy (Microscope Axio Imager Z2, Zeiss, Marly-Le-Roi, France; Metafer, MetaSystems, Altlussheim, Germany). CTCs were defined based on their morphology and specific staining. Expression of N-cadherin is associated with mesenchymal phenotype, cytokeratin is associated with epithelial phenotype, and CD44 is associated with HNSCC stem cell phenotype. Antibody specificity was validated on SQ20-CD44+ cells [18], a subpopulation of CSCs isolated from the HNSCC cell line, SQ20B, that expresses N-cadherin, cytokeratin, and CD44 (Figure 1A). Morphological studies enabled elimination of apoptotic bodies, cell debris, and neutrophilic polynuclear cells. Moreover, CTC is a cell with a round nucleus and a diameter around 20 µm without a real cut-off that can be defined. The use of anti-CD45 antibody specific for leukocytes enabled CD45-free cells to be the focus of our analysis. The combination of both evaluations allowed us to eliminate this population considered as false positive in contrast to the other cells considered as CTCs. CTCs could be positive for one marker and for DAPI and associated with the corresponding phenotype, or positive only for DAPI with an undefined phenotype. Representative images of immunostaining of CTCs are presented in Figure 1B and 1C. Two slides per patient per time point were analyzed. Results were reported as the number of CTCs identified per mL of whole blood. When more than three CTCs were aggregated, they were considered as a cluster, and each cell was counted.

Statistical analysis. Statistical analyses were performed using GraphPad Prism (v.8.4.2, GraphPad Software, San Diego, CA, USA). The association between CTC count and clinical characteristics described in Table 1 was evaluated using Fisher’s exact test. PFS and OS were assessed in the groups stratified according to their clinical response to treatment at 24 months, and the association between changes in CTC count, treatment response, and prognosis were evaluated. Survival rates were assessed using the Kaplan–Meier method. The minimum level of significance was set at *p* < 0.05.

## 3. Results

### 3.1. Counting and Characterization of CTCs 

Before any treatment, 6 out of 21 patients were found to have CTCs (28.6%) (Table 2). The minimum, maximum, and median CTC counts were 0.07 CTC/mL, 3.34 CTC/mL, and 0.22 CTC/mL, respectively. No significant associations were observed between the number of CTCs at baseline and clinical characteristics of the patients, including sex, age, clinical stage (tumor and nodes), tobacco use, alcohol intake, and human papillomavirus status (Table 1). On day 21, before the second course of DCF treatment, CTCs were collected from 11 patients who received DCF chemotherapy, as well as from 1 patient who received mDCF chemotherapy. Among the 12 patients from whom blood was collected at day 21, 5 (41.6%) had CTCs (Table 2).

The characterization of CTCs at baseline identified two patients with an epithelial CTC phenotype (cytokeratin expression), three patients with a mesenchymal CTC phenotype (N-cadherin), but no patients displaying stem cell CD44 expression. The six patients with CTCs also exhibited CTCs with undefined phenotypes. The characterization of CTCs at day 21 identified one patient with an epithelial CTC phenotype, two patients with a mesenchymal CTC phenotype, and one patient with a stem cell phenotype. The five patients with CTCs also exhibited CTCs with undefined phenotypes, including two patients with clusters (Table 2). No significant associations were observed between CTC phenotype at baseline or day 21 and OS or PFS (data not shown). 

### 3.2. Association between the Presence of CTCs at Baseline or Day21 and the Survival Rate

**Figure 2:**
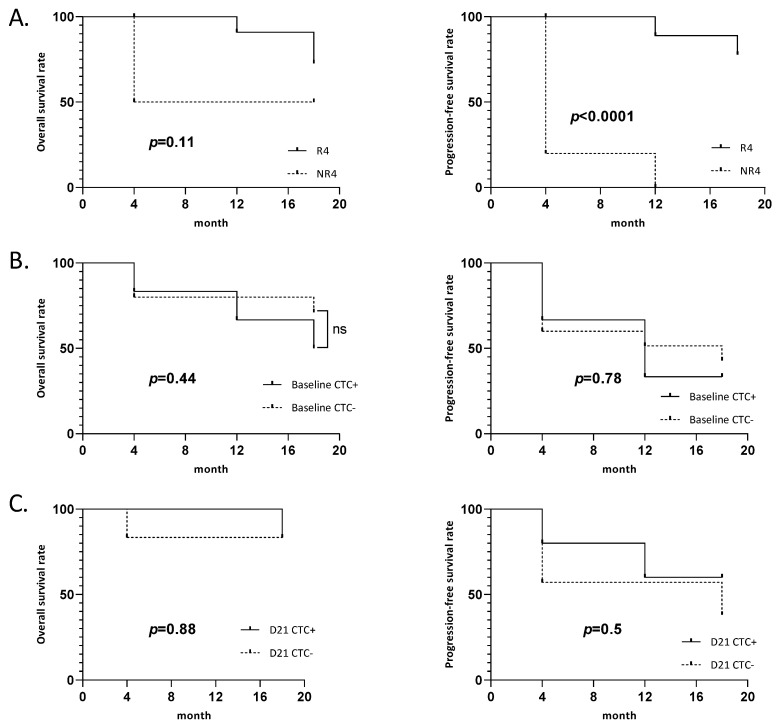
Survival curves: (**A**) Overall survival and progression-free survival of early responder. (R4: responder at 4 months post treatment, NR4: nonresponder at 4 months post treatment). (**B**) Overall survival and progression-free survival of patients depending on CTC at baseline. (**C**) Overall survival and progression-free survival of patients depending on CTC at day 21 (D21).

Of the 21 patients, 8 were considered as early nonresponders at 4 months follow-up. The PFS was significantly lower for early nonresponders compared to responders (*p* < 0.0001, hazard ratio (HR) 30.4; confidence interval (CI), 6.6–139.3), and the OS was not statistically different (*p* = 0.11) (Figure 2A). Regarding CTCs, 4 early responders (patients #1, #2, #3, and #12) and 2 early nonresponders (patients #15 and #20) had CTCs at baseline. On day 21, 3 early responders (patient #2, #3, and #5) and 2 early nonresponders (patients #14 and #15) had CTCs (Table 2). Regardless of responder classification, there was no significant difference in the OS and PFS between patients with or without CTCs at baseline (*p* = 0.44 and 0.78, respectively) or day 21 (*p* = 0.88 and 0.5, respectively) (Figure 2B,C).

### 3.3. Variation in the Number of CTCs between Baseline and D21 and the Survival Rate

**Figure 3:**
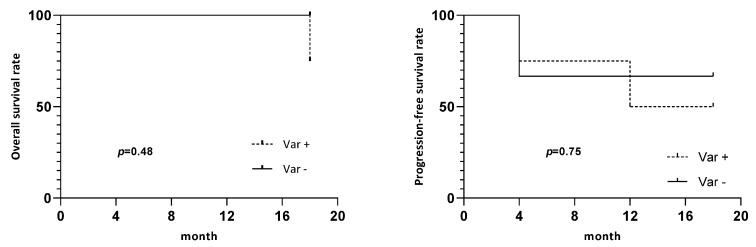
Survival curves. Overall survival and progression-free survival of patients depending on CTC’s variation between baseline and day 21. Var +: positive variation of CTCs, Var -: no positive variation.

Of the 11 DCF patients who had a blood sample collected at day 21, 4 patients (33%) (patients #2, #3, #5, and #14) had a positive variation of CTCs (an increase in CTCs between baseline and day 21) and 2 had clusters at day 21 (patient #3, early responder and patient #14, early nonresponder). Two patients (16.7%) (patients #1 and #15) had a negative variation (decreased CTCs between baseline and day 21). There was no significant association between a positive variation in the CTC number and CTC phenotype or cluster. Despite the absence of a significant difference in the OS and PFS between patients with a positive variation and negative variation in CTCs (*p* = 0.48 and 0.75, respectively) (Figure 3), we observed a clear tendency in patients with a positive variation to have a lower OS. 

## 4. Discussion

We conducted a prospective pilot study to explore the potential role of CTCs during neoadjuvant chemotherapy to predict PFS and OS in patients with oropharyngeal cancer. We showed that there was no significant difference in PFS or OS between patients with and without CTCs at either baseline or day 21, but we observed a variation in the number of detected CTCs in some patients during the first 3 weeks of treatment. Despite the positive variation in CTCs, meaning an increase in CTCs during chemotherapy treatment, the change was not statistically significant because of the relatively low number of patients. Even so, a clear tendency to poor prognosis emerged from the results. 

CTCs have already been evaluated in various cancers, and some studies showed that increased CTCs are correlated with a poorer prognosis [19,20]. A meta-analysis of CTCs in breast cancer indicated that CTC-positive patients (≥5 CTCs/7.5 mL) displayed an increased risk of both tumor progression and death [21]. In a recent review concerning lung cancer, non-small cell lung cancer, and small cell lung cancer, it was shown that an increase in CTCs correlated with a poor prognosis [22]. In a study of 216 patients with ovarian cancer, patients exhibiting ≥ 2 CTCs at baseline presented a decreased PFS and OS [23,24]. 

For HNSCC, few studies explored the role of CTCs before, during, and after treatment, and unfortunately, they are based on small cohorts and different methods for both isolation and counting CTCs. In a cohort of 73 patients with hypo- and oropharynx tumors, Buglione et al. demonstrated that a partial or complete response to chemotherapy was associated with the absence or disappearance of CTCs during treatment. In addition, a decrease in the number of CTCs or their absence during treatment also appeared to be associated with non-progressive disease. Unfortunately, in this study, the authors examined different anatomic subsites of cancer and different histopathological types of cancers such as squamous cell carcinoma or sinonasal undifferentiated carcinoma, which have different clinical outcomes [25]. Another study showed that the presence of CTCs expressing markers such as, cytokeratin, vimentin, EGFR, CD44, or N-Cadherin was correlated with a poor prognosis [26]. In a cohort of 25 patients with oropharynx cancer treated with neoadjuvant chemotherapy, Inhestern et al. showed that there was no correlation between the presence of CTCs and age, sex, tumor site, stage, or lymph node involvement. Furthermore, a high number of CTCs at baseline and after the treatment was proposed as a prognostic marker for OS [12], but an analysis of the correlation between the variations in the numbers of CTCs and PFS and OS was not addressed in this study. 

The results concerning the number of CTCs often vary between studies due to the techniques used. The previously cited studies used CellSearch and flow cytometric assays based on a positive epithelial cell adhesion molecule (EpCAM) expression to isolate CTCs. Currently, only the CellSearch technique from Veridex has received approval from the Food and Drug Administration for clinical use in colorectal, lung, prostate, and breast cancers. The analysis is based on an immunological method that counts CD45-, cytokeratin+, and EpCAM+ cells. However, the EpCAM protein is an epithelial marker normally found in most carcinomas, but is weakly expressed in HNSCC tumors [27]. This explains why few experiments that used this device mention the presence of CTCs in patients with HNSCC. 

Three isolation techniques were compared by Kulasinghe et al. in patients with advanced HNSCC: the CellSearch system, ScreenCell (microfiltration device), and RosetteSep (negative enrichment). They found that CellSearch detected CTCs in 8 out of 43 cases (18.6%), ScreenCell in 13 out of 28 cases (46.4%), and RosetteSep in 16 out of 25 cases (64.0%), the latter being able to also detect CTC clusters [28]. These results confirm that RosetteSep is an appropriate tool for the isolation of CTCs in HNSCC.

Concerning the kinetics of CTCs during treatment, the French multicenter CIRCUTEC study focused on patients with nonoperable or metastatic tumor relapse. Sixty-five patients treated with cetuximab chemotherapy were included. CTCs were isolated and detected by three methods: CellSearch, EPISPOT, and flow cytometry. Patients were tested at baseline and on days 7 and 21. Median PFS time was significantly lower in patients with increasing or stable CTC counts (36/54) from baseline to day 7 with EPISPOT and in patients with one CTC detected with a combination of 2 tests at day 7 [29]. For patients with curative intent, Wang et al. analyzed CTC counts before and during radiochemotherapy treatment in patients with locally advanced HNSCC. CTCs were detected using a negative selection strategy and a flow cytometry protocol. The positive variation in the number of CTCs correlated with lower PFS (and OS) [30]. 

Our results suggest the use of CTC kinetics during treatment is much more relevant than the detection of CTC levels alone. Our results are encouraging because it is important to develop predictive biomarkers for responses to neoadjuvant chemotherapy. Indeed, these treatments are associated with complications (e.g., hematological, renal, and auditory side effects). Thus, the early identification of nonresponding patients through an analysis of the variation in the number of CTCs may allow an early adjustment of the therapeutic strategy. This would improve survival while limiting the side effects of unnecessary treatments. Moreover, we observed that responding patients at 4 months after the end of treatment had significantly better OS and PFS. Four responder patients at 4 months (patients #2, #9, #12, and #20) showed tumor progression in the following weeks. Patient #2 was a clinical and radiological responder but showed tumor progression and died at 12 months. This patient had CTCs at baseline and a positive variation in CTCs on day 21. The variation in CTCs could not be assessed for the other three patients because they received mDCF treatment. Adding the CTC count to clinical and radiological investigations could help to earlier orientate the management of patients.

## 5. Conclusions

Our pilot study offers preliminary results that should be consolidated using a larger prospective study. The results suggest that the evaluation of variations in CTCs could be used as a predictive biomarker during treatment, particularly at the time of the morphological and clinical evaluations performed to assess the response to neoadjuvant chemotherapy. When patients show a good response to the clinical and morphological evaluation, they are referred to adjuvant radiotherapy. Unfortunately, some patients will show tumor progression after radiotherapy with worse survival. Thus, the study of the variation in the number of CTCs and their appearance or disappearance would be useful during treatment to better guide management decisions with possible early changes in strategy. 

## Figures and Tables

**Table 1:** Patients’ characteristics.

Patients’ Characteristics	Number of Patients	*n* CTC+ at Baseline	*p*-Value
**Age (years)**			
<65	16 (76.2%)	5	1
>65	5 (23.8%)	1	
**Gender**			
Male	18 (85.7%)	5	1
Female	3 (14.3%)	1	
**T stage**			
T2	2 (9.5%)	0	0.57
T3	8 (38.1%)	3	
T4	11 (52.4%)	3	
**N stage**			
N0	3 (14.3%)	2	0.18
N+	18 (85.7%)	4	
**Tobacco**			
Exposed	19 (90.5%)	6	1
None	2 (9.5%)	0	
**Alcohol**			
Exposed	7 (33.3%)	3	0.29
None	14 (61.9%)	3	
**HPV status**			
Positive	5 (23.8%)	0	
Negative	13 (61.9%)	4	0.12
Unknown	3 (14.3%)	2	

*n* CTC+ at Baseline: number of patients with CTC at baseline.

**Table 2:** Identification and characterization of CTC patients according to treatment protocol.

Patients		Baseline (Number of Cells)					Day 21 (Number of Cells)					Variation
		Epithelial	Mesenchymal	Stem Cell	Undefined	CTC/ mL	Epithelial	Mesenchymal	Stem Cell	Undefined	CTC/ mL	
**DCF**	**#1**	0	1	0	2	0.315	0	0	0	0	0	**-**
	**#2**	0	1	0	2	0.255	1	1	0	20 *	1.505	**+**
	**#3**	0	0	0	1	0.190	0	0	0	6	0.315	**+**
	**#4**	0	0	0	0	0	0	0	0	0	0	**=**
	**#5**	0	0	0	0	0	0	3	1	15	1.190	**+**
	**#6**	0	0	0	0	0	0	0	0	0	0	**=**
	**#7**	0	0	0	0	0	0	0	0	0	0	**=**
	**#14°**	0	0	0	0	0	0	0	0	38 *	2.375	**+**
	**#15°**	0	0	0	3	0.880	0	0	0	1	0.055	**-**
	**#16°**	0	0	0	0	0	0	0	0	0	0	**=**
	**#17°**	0	0	0	0	0	0	0	0	0	0	**=**
	**#18°**	0	0	0	0	0	/	/	/	/	/	NA
**mDCF**	**#8**	0	0	0	0	0	/	/	/	/	/	NA
	**#9**	0	0	0	0	0	/	/	/	/	/	NA
	**#10**	0	0	0	0	0	0	0	0	0	0	**=**
	**#11**	0	0	0	0	0	/	/	/	/	/	NA
	**#12**	1	0	0	1	0.125	/	/	/	/	/	NA
	**#13**	0	0	0	0	0	/	/	/	/	/	NA
	**#19°**	0	0	0	0	0	/	/	/	/	/	NA
	**#20°**	1	1	0	8	3.335	/	/	/	/	/	NA
	**#21°**	0	0	0	0	0	/	/	/	/	/	NA

DCF: Docetaxel, Cisplatine, 5-Fluorouracil. mDCF: DCF modified (dose adapted). Variation: increase (+), decrease (-) or stable (=) variation in CTCs between baseline and day 21. *: cluster of CTCs. °: early nonresponder patient. /: unmeasured. NA: not applicable.

## Data Availability

Pr Rodriguez-Lafrasse had full access to all the data in the study and takes responsibility for the integrity of the data and the accuracy of the data analysis. The data that support the findings of this study are available upon reasonable request to the corresponding author.

## References

[1] Ferlay J., Soerjomataram I., Dikshit R., Eser S., Mathers C., Rebelo M., Parkin D.M., Forman D., Bray F. (2015). Cancer incidence and mortality worldwide: Sources, methods and major patterns in GLOBOCAN 2012. Int. J. Cancer.

[2] Takes R.P., Rinaldo A., Silver C.E., Haigentz M., Woolgar J.A., Triantafyllou A., Mondin V., Paccagnella D., de Bree R., Shaha A.R. (2012). Distant metastases from head and neck squamous cell carcinoma. Part I. Basic aspects. Oral Oncol..

[3] Routray S., Mohanty N. (2014). Cancer stem cells accountability in progression of head and neck squamous cell carcinoma: The most recent trends!. Mol. Biol. Int..

[4] Yoshida G.J., Saya H. (2021). Molecular pathology underlying the robustness of cancer stem cells. Regen. Ther..

[5] Lianidou E.S., Markou A., Strati A. (2015). The Role of CTCs as Tumor Biomarkers. Adv. Exp. Med. Biol..

[6] Galletti G., Portella L., Tagawa S.T., Kirby B.J., Giannakakou P., Nanus D.M. (2014). Circulating tumor cells in prostate cancer diagnosis and monitoring: An appraisal of clinical potential. Mol. Diagn. Ther..

[7] Giordano A., Egleston B.L., Hajage D., Bland J., Hortobagyi G.N., Reuben J.M., Pierga J.-Y., Cristofanilli M., Bidard F.-C. (2013). Establishment and validation of circulating tumor cell-based prognostic nomograms in first-line metastatic breast cancer patients. Clin. Cancer Res..

[8] Tsai W.-S., Chen J.-S., Shao H.-J., Wu J.-C., Lai J.-M., Lu S.-H., Hung T.-F., Chiu Y.-C., You J.-F., Hsieh P.-S. (2016). Circulating Tumor Cell Count Correlates with Colorectal Neoplasm Progression and Is a Prognostic Marker for Distant Metastasis in Non-Metastatic Patients. Sci. Rep..

[9] Zou K., Yang S., Zheng L., Wang S., Xiong B. (2016). Prognostic Role of the Circulating Tumor Cells Detected by Cytological Methods in Gastric Cancer: A Meta-Analysis. Biomed. Res. Int..

[10] Bozec A., Ilie M., Dassonville O., Long E., Poissonnet G., Santini J., Chamorey E., Ettaiche M., Chauvière D., Peyrade F. (2013). Significance of circulating tumor cell detection using the CellSearch system in patients with locally advanced head and neck squamous cell carcinoma. Eur. Arch. Oto-Rhino-Laryngol..

[11] Grisanti S., Almici C., Consoli F., Buglione M., Verardi R., Bolzoni-Villaret A., Bianchetti A., Ciccarese C., Mangoni M., Ferrari L. (2014). Circulating tumor cells in patients with recurrent or metastatic head and neck carcinoma: Prognostic and predictive significance. PLoS ONE.

[12] Inhestern J., Oertel K., Stemmann V., Schmalenberg H., Dietz A., Rotter N., Veit J., Görner M., Sudhoff H., Junghanß C. (2015). Prognostic Role of Circulating Tumor Cells during Induction Chemotherapy Followed by Curative Surgery Combined with Postoperative Radiotherapy in Patients with Locally Advanced Oral and Oropharyngeal Squamous Cell Cancer. PLoS ONE.

[13] Jatana K.R., Balasubramanian P., Lang J.C., Yang L., Jatana C.A., White E., Agrawal A., Ozer E., Schuller D.E., Teknos T.N. (2010). Significance of circulating tumor cells in patients with squamous cell carcinoma of the head and neck: Initial results. Arch. Otolaryngol. Head Neck Surg..

[14] Winter S.C., Stephenson S.-A., Subramaniam S.K., Paleri V., Ha K., Marnane C., Krishnan S., Rees G. (2009). Long term survival following the detection of circulating tumour cells in head and neck squamous cell carcinoma. BMC Cancer.

[15] Tang K.D., Vasani S., Taheri T., Walsh L.J., Hughes B.G.M., Kenny L., Punyadeera C. (2020). An Occult HPV-Driven Oropharyngeal Squamous Cell Carcinoma Discovered Through a Saliva Test. Front. Oncol..

[16] Ekanayake Weeramange C., Liu Z., Hartel G., Li Y., Vasani S., Langton-Lockton J., Kenny L., Morris L., Frazer I., Tang K.D. (2021). Salivary High-Risk Human Papillomavirus (HPV) DNA as a Biomarker for HPV-Driven Head and Neck Cancers. J. Mol. Diagn..

[17] Hodgkinson C.L., Morrow C.J., Li Y., Metcalf R.L., Rothwell D.G., Trapani F., Polanski R., Burt D.J., Simpson K.L., Morris K. (2014). Tumorigenicity and genetic profiling of circulating tumor cells in small-cell lung cancer. Nat. Med..

[18] Moncharmont C., Guy J.-B., Wozny A.-S., Gilormini M., Battiston-Montagne P., Ardail D., Beuve M., Alphonse G., Simoëns X., Rancoule C. (2016). Carbon ion irradiation withstands cancer stem cells’ migration/invasion process in Head and Neck Squamous Cell Carcinoma (HNSCC). Oncotarget.

[19] Kulasinghe A., Zhou J., Kenny L., Papautsky I., Punyadeera C. (2019). Capture of Circulating Tumour Cell Clusters Using Straight Microfluidic Chips. Cancers.

[20] Kulasinghe A., Schmidt H., Perry C., Whitfield B., Kenny L., Nelson C., Warkiani M.E., Punyadeera C. (2018). A Collective Route to Head and Neck Cancer Metastasis. Sci. Rep..

[21] Moussavi-Harami S.F., Wisinski K.B., Beebe D.J. (2014). Circulating Tumor Cells in Metastatic Breast Cancer: A Prognostic and Predictive Marker. J. Patient Cent. Res. Rev..

[22] Kapeleris J., Kulasinghe A., Warkiani M.E., Vela I., Kenny L., O’Byrne K., Punyadeera C. (2018). The Prognostic Role of Circulating Tumor Cells (CTCs) in Lung Cancer. Front. Oncol..

[23] Poveda A., Kaye S.B., McCormack R., Wang S., Parekh T., Ricci D., Lebedinsky C.A., Tercero J.C., Zintl P., Monk B.J. (2011). Circulating tumor cells predict progression free survival and overall survival in patients with relapsed/recurrent advanced ovarian cancer. Gynecol. Oncol..

[24] Zhang X., Li H., Yu X., Li S., Lei Z., Li C., Zhang Q., Han Q., Li Y., Zhang K. (2018). Analysis of Circulating Tumor Cells in Ovarian Cancer and Their Clinical Value as a Biomarker. Cell Physiol. Biochem..

[25] Buglione M., Grisanti S., Almici C., Mangoni M., Polli C., Consoli F., Verardi R., Costa L., Paiar F., Pasinetti N. (2012). Circulating Tumour Cells in locally advanced head and neck cancer: Preliminary report about their possible role in predicting response to non-surgical treatment and survival. Eur. J. Cancer.

[26] Balasubramanian P., Lang J.C., Jatana K.R., Miller B., Ozer E., Old M., Schuller D.E., Agrawal A., Teknos T.N., Summers T.A. (2012). Multiparameter analysis, including EMT markers, on negatively enriched blood samples from patients with squamous cell carcinoma of the head and neck. PLoS ONE.

[27] Kulasinghe A., Hughes B.G.M., Kenny L., Punyadeera C. (2019). An update: Circulating tumor cells in head and neck cancer. Expert Rev. Mol. Diagn..

[28] Kulasinghe A., Kenny L., Perry C., Thiery J.-P., Jovanovic L., Vela I., Nelson C., Punyadeera C. (2016). Impact of label-free technologies in head and neck cancer circulating tumour cells. Oncotarget.

[29] Garrel R., Mazel M., Perriard F., Vinches M., Cayrefourcq L., Guigay J., Digue L., Aubry K., Alfonsi M., Delord J.-P. (2019). Circulating Tumor Cells as a Prognostic Factor in Recurrent or Metastatic Head and Neck Squamous Cell Carcinoma: The CIRCUTEC Prospective Study. Clin. Chem..

[30] Wang H.-M., Wu M.-H., Chang P.-H., Lin H.-C., Liao C.-D., Wu S.-M., Hung T.-M., Lin C.-Y., Chang T.-C., Tzu-Tsen Y. (2019). The change in circulating tumor cells before and during concurrent chemoradiotherapy is associated with survival in patients with locally advanced head and neck cancer. Head Neck.

